# Enhancing motor imagery detection efficacy using multisensory virtual reality priming

**DOI:** 10.3389/fnrgo.2023.1080200

**Published:** 2023-04-06

**Authors:** Reza Amini Gougeh, Tiago H. Falk

**Affiliations:** Institut National de la Recherche Scientifique-Energy, Materials and Telecommunications Center, University of Québec, Montreal, QC, Canada

**Keywords:** brain-computer interface, motor imagery, multisensory priming, virtual reality, haptics, force feedback, olfaction

## Abstract

Brain-computer interfaces (BCI) have been developed to allow users to communicate with the external world by translating brain activity into control signals. Motor imagery (MI) has been a popular paradigm in BCI control where the user imagines movements of e.g., their left and right limbs and classifiers are then trained to detect such intent directly from electroencephalography (EEG) signals. For some users, however, it is difficult to elicit patterns in the EEG signal that can be detected with existing features and classifiers. As such, new user control strategies and training paradigms have been highly sought-after to help improve motor imagery performance. Virtual reality (VR) has emerged as one potential tool where improvements in user engagement and level of immersion have shown to improve BCI accuracy. Motor priming in VR, in turn, has shown to further enhance BCI accuracy. In this pilot study, we take the first steps to explore if multisensory VR motor priming, where haptic and olfactory stimuli are present, can improve motor imagery detection efficacy in terms of both improved accuracy and faster detection. Experiments with 10 participants equipped with a biosensor-embedded VR headset, an off-the-shelf scent diffusion device, and a haptic glove with force feedback showed that significant improvements in motor imagery detection could be achieved. Increased activity in the six common spatial pattern filters used were also observed and peak accuracy could be achieved with analysis windows that were 2 s shorter. Combined, the results suggest that multisensory motor priming prior to motor imagery could improve detection efficacy.

## 1. Introduction

Brain-computer interfaces (BCI) represent a burgeoning modality to control and communicate with peripheral devices via non-muscular motor control and directly through brain signals (Vidal, 1973). To establish a dialogue between the brain and computers, the first BCI systems were developed based on electroencephalography (EEG) signals in the early 1970s (Wolpaw et al., 2002). Their primary goals focused on clinical applications to repair, improve, or replace the natural output of the human central nervous system (Wolpaw and Wolpaw, 2012). Today, so-called passive/affective BCIs have emerged to monitor unintentional, involuntary, and spontaneous modulations in user cognitive states (Zander and Jatzev, 2011). For example, BCIs have been proposed to detect emotion states during meditation (Kosunen et al., 2017), to provide insights on a user's quality of experience (Gupta et al., 2016), and have recently been integrated into virtual reality headsets to allow for personalized experiences (Bernal et al., 2022; Moinnereau et al., 2022), just to name a few applications.

Reactive BCI systems, in turn, are driven by explicit communication between humans and computers by exploiting brain activity arising in reaction to external stimulation, which is indirectly modulated by the user to control an application (Wolpaw et al., 2020). For example, spellers using the P300 event-related potential (ERP) have been proposed and perfected over the years (e.g., Philip and George, 2020), as have games based on steady-state visual evoked potentials (SSVEP) (e.g., Lopez-Gordo et al., 2019; Filiz and Arslan, 2020). Active BCIs, on the other hand, derive their outputs from brain activity which is directly and consciously controlled by the user, independent of external events, to control an application. The most common mental task used is motor imagery (MI), where the person imagines moving their hands, feet, and/or tongue and these imagined movements produce modulations over the sensorimotor cortex.

MI-based BCIs are very popular (Zhang and Wang, 2021) as they have shown to engage the same underlying neural circuits associated with executed motor actions (Abbruzzese et al., 2015). As such, they have been used to control, for example, wheelchairs, drones, and exoskeletons (Kim et al., 2016) or to improve attention levels (Yang et al., 2018) in both healthy and patient populations (Ruffino et al., 2017). In the medical field, they have been employed for stroke rehabilitation (Tang et al., 2018). In this case, the coupling of the BCI with a functional electrical stimulator allows for direct feedback to the user via muscle stimulation (Marquez-Chin and Popovic, 2020) once a successful imagery task is achieved. Research is showing that when the MI task is repeated many times, it can induce greater brain plasticity, reduce spasticity, and help patients more quickly restore movements (Sebastián-Romagosa et al., 2020).

Despite these reported benefits of using MI-based BCIs, studies have reported that detecting motor imagery tasks using off-the-shelf neuroimaging tools can be challenging for 15–30% of the population (Blankertz et al., 2010; Thompson, 2019). Earlier studies referred to this as “BCI illiteracy,” which insinuates the issue is on the user. While, indeed, studies have shown that MI-BCI accuracy can be affected by user-related factors, such as attention and frustration levels (Myrden and Chau, 2015), limitations in hardware (e.g., signal acquisition systems and signal quality) and software (e.g., accuracy of the classification algorithms) also play a crucial role (Allison and Neuper, 2010). As such, the terminology “BCI inefficiency” (Edlinger et al., 2015) has been more recently incorporated. To overcome this issue, recent research has focused on developing new filtering methods, feature extraction techniques, and newer and more complex machine learning algorithms to tackle the software aspect (Gaur et al., 2015; Zhou et al., 2020; Benaroch et al., 2022; Tibrewal et al., 2022). Moreover, improvements in bioamplifiers and electrodes (dry versus gel-based; active versus passive) have been explored to address the hardware issues (Cecotti and Rivet, 2014). Lastly, new training paradigms and presentation modalities (e.g., virtual reality), as well as priming methods have been explored to help users generate neural signals that can be better detected with existing technologies (Birbaumer et al., 2013).

Virtual reality (VR) has emerged as one particular presentation modality that has shown to positively impact BCI performance (Vourvopoulos et al., 2016; Amini Gougeh and Falk, 2022a; Arpaia et al., 2022; Choy et al., 2022). VR-based presentation can increase the sense of embodiment, improve immersion, and foster greater engagement levels, which, in turn, could lead to the generation of neural signals with increased discriminability (Škola and Liarokapis, 2018; Vourvopoulos et al., 2019). In fact, Amini Gougeh and Falk (2022a) surveyed the literature and showed that VR coupled with MI-BCIs could lead to improved neurorehabilitation outcomes.

In addition to VR-based training, priming strategies have also been explored as a tool to further enhance motor imagery. For instance, Vourvopoulos et al. (2015) and Vourvopoulos and Bermúdez i Badia (2016) showed that motor priming, where a VR-based physical activity was performed prior to the MI task, could enhance the imagery task. In particular, subjects who received motor priming in VR had higher MI-BCI performance compared to a standard setup. Stoykov and Madhavan (2015), in turn, highlighted the potential of sensory priming and showed how incorporating vibration stimuli during priming could improve motor imagery and neurorehabilitation outcomes. Vibrotactile stimuli, however, covers only one modality in sensory priming, which leaves the question “Are there any benefits to including other sensory modalities during VR-based priming?” still unanswered. In this work, we wish to explore the impact of including olfactory and tactile stimuli on overall motor imagery performance in order to help answer this question.

It is known that smells can influence user behavior through affective priming (Smeets and Dijksterhuis, 2014). Multisensory stimuli can result in experiences that enhance the sense of embodiment, presence, immersion, engagement, and overall experience of the user relative to a conventional audio-visual experience (e.g., Melo et al., 2020; Amini Gougeh and Falk, 2022b; Amini Gougeh et al., 2022). As such, it is hypothesized that a multisensory immersive priming paradigm will further improve motor imagery detection accuracy. In fact, olfaction has been linked to improved relaxation states, increased attention, and more positive emotions (Gougeh et al., 2022; Lopes et al., 2022). Hence, inclusion of smells during priming may counter the negative effects that attention and frustration can have on motor imagery accuracy, as reported by Myrden and Chau (2015).

Moreover, sensory processing, including olfactory, has been shown to influence motor processing (Ebner, 2005). In a recent study using transcranial magnetic stimulation, the perception of a pleasant smell and its olfactory imagery showed to be associated with primary motor cortex excitability (Infortuna et al., 2022). The results were in line with those of Rossi et al. (2008), which showed a cross-link between the olfactory and motor systems. The work by Tubaldi et al. (2011), in turn, suggested a superadditive effect on brain activity during action observation executed with a small odorant object (e.g., grasping a strawberry, almond, orange, or apple). Combined, these findings suggest that the combination of motor tasks in the presence of olfactory stimuli could have an impact on user behavior and on motor cortex excitability. Ultimately, this could lead to improved MI-BCI performance.

In light of these insights, our main research question (RQ) is to investigate if VR training, combined with multisensory priming, can improve MI-BCI performance. Here, improvement will be explored across two dimensions: accuracy and timing. Improvements in accuracy suggest neural signatures that are more discriminative for existing signal processing and machine learning pipelines. Improvements in timing suggest that imagery can be detected faster. Timing is important, as it is known that mental fatigue from long task times can induce frustration and, ultimately, reduce overall BCI performance (Talukdar et al., 2019). In addition to this main RQ, several other sub-RQs will be explored. These will investigate aspects, such as, user experience and sense of presence provided by the multisensory priming paradigm, the differences in accuracy achieved on a per-subject versus global scale, the impact of varying distances and depths cues of the movement imagination on overall accuracy, as well as the impact of multisensory priming on different aspects of the signal processing pipelines, including window duration size and time-from-cue, and their impact on overall accuracy.

To help answer these questions, a multisensory motor priming experiment was performed in VR. Two motor priming tasks took place between two motor imagery tasks: a multisensory one that included tactile, olfactory, and audio-visual stimuli, and a baseline that included only audio-visual. Different analyses are performed to help answer the RQs and sub-RQs mentioned above. The remainder of this paper is organized as follows. In Section 2, we detail the material and methods used in the study. Experimental results are then presented in Section 3 and discussed in light of existing literature. Lastly, Conclusions are presented in Section 4.

## 2. Materials and methods

### 2.1. Participants

Eleven participants (three female, 25.81 ± 3.88 years old) were recruited to participate in this pilot study. Eligibility criteria included healthy individuals. Participants with a history of severe cybersickness and sensitivity to scents were excluded. The experiment protocol was reviewed and approved by the Ethics Committee of the Institut national de la recherche scientifique (INRS), University of Quebec (number: CER-22-663). During data collection, COVID-19 safety measures were considered and put in place, including maintaining social distance, wearing a face mask, and disinfecting all devices with alcohol wipes and a UV-C chamber. All participants were considered novice BCI users, and this was their first time performing a motor imagery task. It is important to emphasize that the data from one subject was considered too noisy for analysis, thus was discarded here from the analysis.

### 2.2. Equipment and data integration

**Figure 1:**
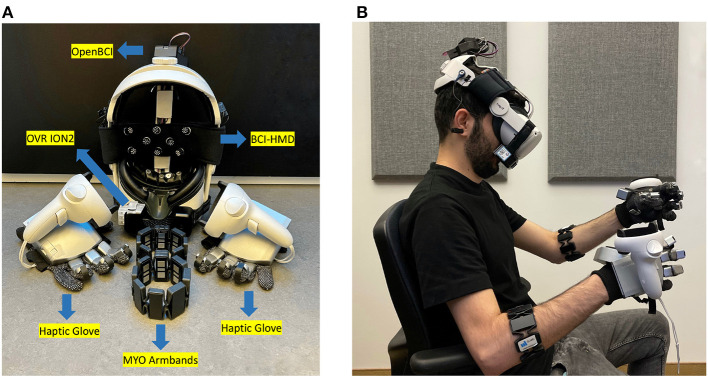
**(A)** Instrumented BCI-HMD headset comprised of a HMD-VR and bioamplifier to monitor electroencephalography (EEG), electrooculography (EOG), facial electromyography (EMG), and photoplethysmography (PPG). Other devices include the OVR ION2 scentware, Myo armbands, and a Senseglove Nova haptic glove. **(B)** A participant wearing the BCI-HMD system.

In this study, a VR head-mounted display (HMD) was coupled with force feedback haptic gloves, an electromyogram (EMG) armband, a portable scent diffusion device, and a wireless BCI system (henceforth referred as “BCI-HMD") embedded directly into the headset following guidelines described by Cassani et al. (2020). An illustration of the different components of the system is shown in Figure 1A, along with a visual of a user wearing them in Figure 1B. In addition, a VR game was created and synchronized with the hardware. More details about the instrumented HMD is given next.

#### 2.2.1. Instrumented headset: BCI-HMD

A Meta Quest2 HMD (LCD display with a resolution of 1, 920×1, 832, 72 Hz refresh rate, and 89° field of view) was used. Three physiological signal modalities, including electroencephalography (EEG), electrooculography (EOG), and photoplethysmography (PPG) were integrated into the facial foam and head straps of the VR headset and directly connected to an OpenBCI bioamplifier encased in a 3D-printed box and placed on top of the HMD straps (see Figure 1B). OpenBCI Cyton and Daisy bioamplifiers (OpenBCI, USA) were used to capture 11 EEG channels from frontal (Fp1, Fpz, Fp2, F3, F4, Fz, Fc1, Fc2) and central (C3, C4, Cz) regions following the international 10-20 system (Jasper, 1958) at a 125 Hz sample rate. In order to ensure participant comfort, softPulse^*TM*^ soft dry EEG electrodes (Datwyler, Switzerland) were employed on the VR strap and flat sensors were placed directly on the faceplate cushion of the headset. A PPG sensor was used to monitor heart rate and was also integrated into the faceplate of the HMD, as well as two pairs of vertical and horizontal EOG electrodes. A green LED light was used in the PPG sensor to ensure more accurate measurements (Castaneda et al., 2018). Moreover, a pair of Myo 8-channel armbands (Thalmic labs, Canada) were placed on the participant forearms in order to capture electromyography (EMG) signals at a rate of 200 Hz using dry electrodes. Different modalities and signal inputs were synchronized and recorded using lab streaming layer (LSL) to be used in post-experiment offline data processing.

#### 2.2.2. Multisensory stimuli

A pair of Nova^*TM*^ haptic gloves (SenseGlove, Netherlands) were employed to deliver accurate force feedback to each finger. The gloves could also track wrist, hand, and finger gestures using inertial measurement unit (IMU) sensors. Taking advantage of linear resonant actuators on the thumb and index fingers, participants could perceive the texture and stiffness of a 3D object in the virtual environment. Mounting the Meta Quest2 controllers on the gloves enabled 3D mapping of the hand locations onto the virtual space, as shown in Figure 1B. Furthermore, an OVR ION2 scentware device (OVR Technologies, USA) was connected to the BCI-HMD to provide an olfactory stimulus by dispersing aromas close to the user's nose. The employed scent kit contains nature-oriented scents including beach, flowers, earth dirt, pine forest, ocean breeze, wood, citrus, ozone, and grass smells. In this study, the citrus scent was chosen as an olfactory stimulus.

#### 2.2.3. Developed virtual environment

**Figure 2:**
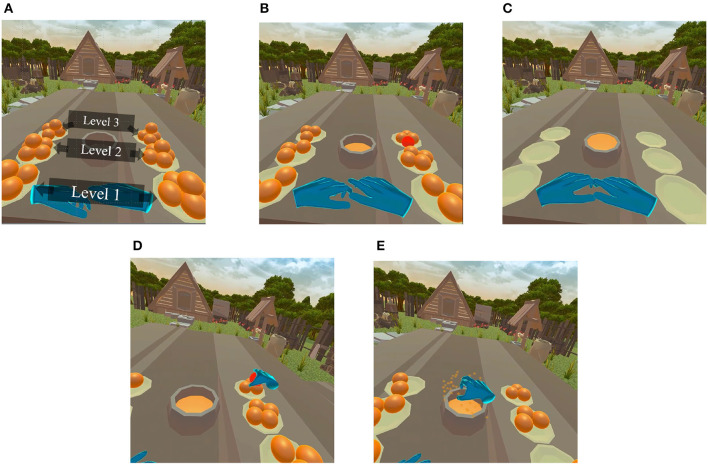
**(A)** Developed game environment, five oranges placed in each of the 6 plates (overall 30 oranges). **(B)** A randomly cued orange in the MI session. The task in the MI session was to imagine grabbing a cued orange, moving it on top of the bowl placed in center, and squeezing it. **(C)** After a pre-determined amount of time, the bowl fills up with orange juice. **(D)** A randomly cued orange that participants were required to grab, move to the top of the center bowl, and squeeze. In the multisensory condition, participants felt the volume and texture of the 3D object, while in audio-visual, oranges could be grabbed and squeezed without any force. **(E)** After successfully squeezing the orange, the cued orange disappeared simultaneously with playing an animation. In the multisensory condition, this event was synchronized with the olfactory stimulus, whereas in the audio-visual condition, it was followed by auditory effects.

A custom virtual environment was designed in Unity3D 2021. As illustrated in Figure 2A, five oranges were placed on each of six plates positioned on top of a table. The participant was seated at the center and three plates to the left and three to the right side of the participant were strategically positioned at three different distances (three levels). The nearest plates (level 1) positioned at a relative distance of 20 cm from subject in the real-world, while the farthest plates (level 3) were located at 60 cm; the middle plates were placed at 40cm (level 2). With this arrangement, we could investigate the role of depth cues on sense of presence and, ultimately, on BCI performance. The environment and the distance were devised in a way that even the oranges placed on the farthest plate were accessible by full arm extension, without the need for any additional body movement.

### 2.3. Experimental design

**Figure 3:**
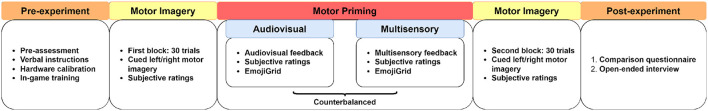
An overview of the experiment timeline. Screening for motion sickness and smell sensitivity was part of the pre-assessment process. Afterwards, instructions were given, followed by system calibration and in-game training. The experiment begun with a motor imagery session, where users only receive visual cues as to which oranges to grab and when the imagery task was concluded. Subsequently, the motor priming block consisted of two experimental conditions with varying types of sensory stimuli. Next, another motor imagery session was conducted. A final post-experiment interview was conducted.

A repeated measures experimental design (so-called within-subject experimental protocol) was followed in which all the participants underwent the same experimental conditions (with counterbalanced ordering). Figure 3 illustrates the step-by-step protocol timelines and experimental blocks. In the pre-experiment phase, participants were first assessed for any known motion sickness or sensitivity to smells, as well as their comfort with the BCI-HMD and haptic gloves. Each participant was then given comprehensive instructions verbally, before wearing the Myo armbands and BCI-HMD. Afterwards, all the systems were calibrated, signal quality checked, and any adjustments were made to increase signal quality. Finally, participants received in-game training with and without multisensory stimuli.

After the pre-experiment, participants were asked to perform a common BCI MI task where they imagined grabbing an orange using either their left or right hand (Chatterjee and Bandyopadhyay, 2016). In this MI session, the user was cued to imagine reaching for and grabbing the orange that has turned red for a duration of 10 s. Oranges were randomly turned red between the left and right sides, as shown in Figure 2B. During the 10 s, the participants were instructed to imagine grabbing the cued orange, moving it to the middle of the table, and squeezing it onto the center bowl. After the 10 s the orange disappeared for a 5-s rest period, after which another orange was cued. This procedure was repeated until all 30 oranges (5 oranges × 6 plates) were squeezed, all plates became empty, and the center bowl filled with orange juice, as depicted by Figure 2C). It is important to emphasize that this was an offline MI-BCI task, without any online BCI classification model involved. The visuals were provided only as cues to which orange to imagine grabbing and when to finalize the imagery task.

Following this first motor imagery task, participants were instructed to perform the motor priming (MP) task twice, once with only audio-visual stimuli (audiovisual MP) and once with the multisensory (multisensory MP) stimuli. A counterbalanced ordering of conditions was applied across subjects to eliminate any biases. Given the high inter-subject variability of MI-BCI accuracy, as reported previously (Blankertz et al., 2010; Thompson, 2019), it was decided to have all participants perform both tasks, as opposed to having half of them perform the multisensory MP task and the other half the conventional task. The work by Wriessnegger et al. (2018) showed the benefits of motor execution training prior to MI. As such, we will refer to the condition preceding the last MI task as the motor priming condition in our analyses and compare the conventional and multisensory MP paradigms.

In the motor priming sessions, a randomly assigned orange was cued in red color and users were instructed to grab them (Figure 2D), move them over the center bowl, and squeeze the orange until all juice was extracted, causing the orange to burst and vanish and the juice level in the bowl to rise (Figure 2E). Participants had their real-world hand movement mapped onto the virtual hand models (shown as blue hands in the figures) with a 100 Hz refresh rate. Unlike the MI sessions where imagery was performed for a fixed period of time (i.e., 10 s), in the motor priming sessions participants were allowed to interact with the virtual environment at their own pace and a trial ended only when the orange was successfully squeezed. In the audiovisual MP experimental condition, squeezing an orange was followed by a “squish” sound effect synced with an animation without applying any physical force feedback. In the multisensory MP condition, on the other hand, users were able to feel the 3D shape and texture of the oranges in the virtual environment and had to apply force in order to successfully squeeze and extract the orange juice. In this condition, the audio-visual stimulus was also time-aligned with a 3-s burst of citrus scent. The experiment concluded when all oranges had been squeezed and all juice extracted.

After the two counterbalanced priming conditions were performed, users underwent a second (offline) motor imagery task following the same instructions and procedure as the first MI session described above. All the participants completed both training conditions, however, half of them completed the audiovisual MP first, whereas the other half completed the multisensory MP task first. Upon completion of each priming condition, participants responded to several questionnaires that appeared directly into the game environment (more details in the next section) as well as the EmojiGrid, a graphical tool to gauge user emotions. At the end, a post-experiment comparison questionnaire was answered and an open-ended interview was conducted.

### 2.4. Subjective ratings

After completion of each priming condition, participants answered questions about their quality of experience, including their perceived level of presence, immersion, realism, engagement, overall experience, and cybersickness using a 5-point absolute category rating like scale ranging from low/poor to high/excellent (ITU, 2008). The ratings and questions were embedded directly into the game environment to avoid any disruption in the experience; the toolkit developed by Feick et al. (2020) was used. Moreover, subjects also reported their valence and arousal state levels using the Emojigrid (Toet and van Erp, 2019), a graphical instrument used to capture emotional states on a continuous Cartesian grid. After both MI sessions, participants also rated their perceived difficulty in performing each MI task using a 5-point scale with options “very easy,” “easy,” “neutral,” “difficult,” and “very difficult.” At the end of the overall experiment, users were asked to rate their preference for the audio-visual or multisensory MP conditions in terms of presence, immersion, realism, engagement, and overall experience. Lastly, an open-ended interview was conducted to gather candid feedback from the participants.

### 2.5. Signal processing and classification

Physiological data including EEG, EOG, EMG, and PPG signals were synchronized with game events using lab stream layer (LSL). In this paper, we focused only on the EMG and EEG signals. Using MATLAB (R2021a, The MathWorks, USA), the EMG signal was band-pass filtered between 10 and 500 Hz and full-wave rectified. The mean absolute value (MAV) of the EMG signal was then extracted for both left and right arms, as suggested by Alkan and Günay (2012), and finally averaged across arms to obtain a final overall muscle activity for each user.

Using the EEGLAB toolbox (Delorme and Makeig, 2004), the EEG signals were band-pass filtered between 4 and 70 Hz, then zero-mean normalized, and spectral power from alpha (7–13 Hz) and beta (13–30 Hz) bands were computed, as suggested by Ahn et al. (2013). Artifact subspace reconstruction (ASR) was also employed to eliminate movement-related artifacts (Kothe and Jung, 2016). For offline classification, common spatial pattern (CSP) features were extracted from the MI trials (Nguyen et al., 2018; Jiang et al., 2020; Amini Gougeh et al., 2021) and fed to a support vector machine (SVM) classifier to discriminate between left or right hand motor imagery (Ramoser et al., 2000; Dornhege et al., 2005).

For binary tasks, CSP features calculate spatial filters that maximize the variance of one class while simultaneously minimizing the variance for the other class. The spatially filtered signal *S* of an EEG trial is given by:


(1) $$\begin{array}{l} S_{L\times T}=W_{L\times N}\times M_{N\times T}, \end{array}$$

where *W* is a *L* × *N* matrix of spatial filters, whereas *L* is the number of filters and *N* number of EEG channels. *M* represents the EEG signal of a certain trial with *N* rows and *T* data points. The first *J* rows of the *W* matrix reflect the maximum variance in the first class (and minimum variance in the second class) and the last *J* rows reflect maximum variance in second class. In this study, we used six spatial filters, three (*J* = 3) from each side, as suggested by Blankertz et al. (2007).

Here, several tests were performed. First, we explored the accuracy achieved with CSP features computed over the entire 10-s (cue duration) motor imagery trial. Next, we explored the use of different window sizes to gauge if priming resulted in motor imagery patterns that could be more quickly discriminated by the available processing pipeline. Lastly, using a fixed window of 5 s duration (as per suggestions by Mzurikwao et al., 2019; Garcia-Moreno et al., 2020; Leon-Urbano and Ugarte, 2020), we explored the impact of different post-task-cue starting points on overall accuracy. As the task involved reaching for an orange, grabbing the orange, moving the orange to the center of the table and squeezing, this analysis could shed some light on what parts of the task may be more discriminative, thus resulting in shorter experimental protocols in the future. These analyses were performed per subject and then averaged to obtain an overall MI-BCI accuracy.

Classification was performed under three settings: in the first, all plates on the left and all plates on the right were aggregated into two classes: left and right. In the second, classification of left versus right imagery was done per level (i.e., plate distance, as shown in Figure 2A) to gauge if depth imagery played a role on motor imagery accuracy. For these two methods, a per-subject analysis was performed and a bootstrap methodology was applied to account for the small dataset size. As such, of the available 30 trials, 20 were randomly assigned to the training set and 10 were set aside for testing and this resampling was repeated 200 times for each subject. An SVM classifier with a linear kernel and default parameters (with *OptimizeHyperparameters* option in MATLAB) was used for classification, resulting in 200 accuracy values per subject which were then used for significance testing. Lastly, in the third setting we pooled all participants together to explore the impact of motor priming on a generic BCI combining neural patterns consistent across multiple subjects. In this case, a total of 300 trials were available (150 left-side oranges and 150 right-side). As done previously, a bootstrap methodology was applied. In this case, 70% of the data was assigned to the training set and 30% to the test set and this was repeated 200 times. An SVM classifier with a linear kernel and default parameters (with *OptimizeHyperparameters* option in MATLAB) was used for classification, resulting in a total of 200 total accuracy values, which were then used for significance testing.

To test the significance of the differences of the two priming conditions, statistical analysis was performed using IBM SPSS 20. Normality of the variables was assessed using the Shapiro-Wilk (S-W) normality test, recommended for small sample size datasets (Elliott and Woodward, 2007). For measures found to be normally distributed, the paired sample t-test was used to determine whether there were statistically significant differences between the two priming conditions. For measures that did not exhibit a normal distribution, a Wilcoxon signed-rank test was performed. We report the obtained mean (M) and standard deviation (SD) across the 200 runs, as well as the p-value. For all analyses, a probability level of *p* < 0.05 was considered to be statistically significant.

## 3. Results and discussion

### 3.1. Subjective ratings

**Figure 4:**
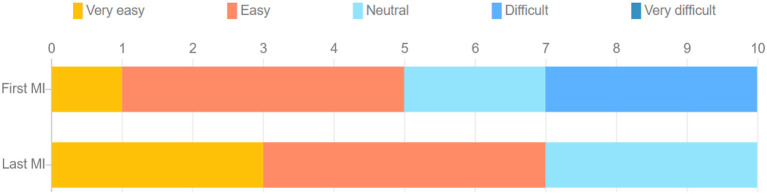
Stacked bar chart of comparison of difficulty of performing MI task during the first and last MI sessions.

**Figure 5:**
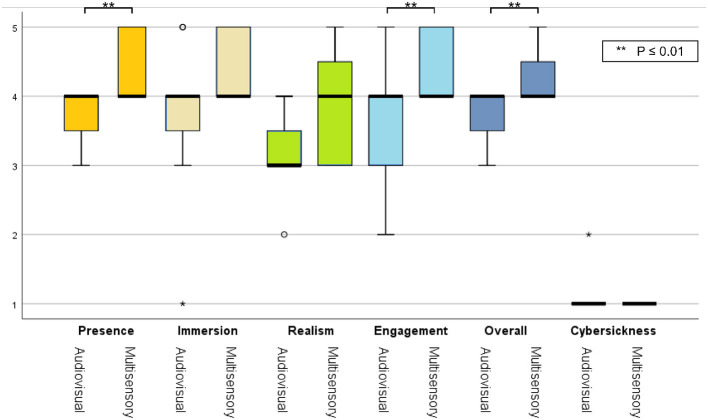
Box plots of subjective ratings averaged across participants for both priming conditions. Asterisks represent statistically significant difference.

The stacked bar chart in Figure 4 depicts the perceived difficulty ratings reported by each subject after execution of the MI tasks. The S-W test indicated a non-normal distribution of these ratings. A statistically significant difference was observed across the first (M = 2.82, SD = 1.079) and the last (M = 2.00, SD = 0.775) MI session (Z = –2.165, *p* = 0.030), suggesting the latter task was perceived as being easier relative to the first. This was expected, as motor imagery tasks have been reported to become easier with training, especially in VR (Wang et al., 2019). For the first MI task, four participants rated it as “easy,” whereas three others rated it “difficult." In the last MI task, on the other hand, all participants rated the task as either “very easy," “easy," or “neutral." Figure 5 depicts bar plots of the average subjective ratings provided after each priming condition. The S-W test indicated a non-normal distribution of subjective ratings. The descriptive statistics observed for each motor priming condition are reported in Table 1.

**Table 1:** Descriptive statistics of the subjective ratings for each motor priming condition.

**QoE subscale**	**Audiovisual MP**	**Multisensory MP**
Presence	3.73 ± 0.467	4.36 ± 0.505
Immersion	3.73 ± 1.104	4.36 ± 0.505
Realism	3.18 ± 0.603	3.73 ± 0.467
Engagement	3.55 ± 0.934	4.45 ± 0.52
Cybersickness	1.09 ± 0.302	1.00 ± 0.000
Overall experience	3.82 ± 0.405	4.27 ± 0.467

Values are mean ± standard deviation.

Using a Wilcoxon signed-rank test, a statistically significant difference was observed for the presence (Z= 2.449, *p*= 0.014), engagement (Z = 1.983, *p*= 0.047) and overall experience (Z = 2.121, *p*= 0.034) ratings; the multisensory MP condition resulted in higher ratings. As the virtual environment was stationary, all participants reported a low cybersickness level and only one participant reported mild dizziness symptoms during the audio-visual session. Lastly, from the EmojiGrid ratings, a statistically significant increase was reported in valence [*t*(9) = 2.882, *p* = 0.018] and arousal [*t*(9) = 7.258, *p* = 0.001] with the multisensory MP condition relative to the audio-visual condition, thus suggesting a more pleasant and exciting experience.

### 3.2. MI-BCI performance per subject

**Figure 6:**
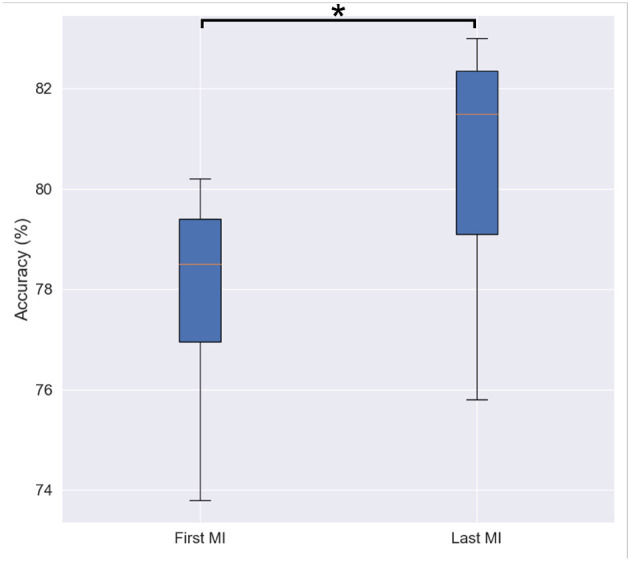
Box-plot of binary classification accuracy for first and last motor imagery sessions. Asterisk shows statistically significant difference among two conditions revealed by a paired sample *t*-test.

As previously described, each MI trial consisted of 10 s of imagery followed by 5 s of rest. Participants were asked to perform the MI task 15 times for all of the left oranges and 15 times for all of the right oranges, totalling 30 trials per subject. Figure 6 depicts the accuracy (averaged across participants and bootstrap instances) achieved for the first and last MI tasks. As can be seen, a statistically significant difference was observed by the paired sample *t*-test between the initial (M = 77.76, SD = 2.23) and the final (M = 80.32, SD = 2.62) MI sessions [*t*(9)= 2.407, *p*= 0.039]. This finding, aligned with the subjective reports described above, highlights the importance of priming prior to the motor imagery task, thus corroborating the findings from Daeglau et al. (2020).

**Figure 7:**
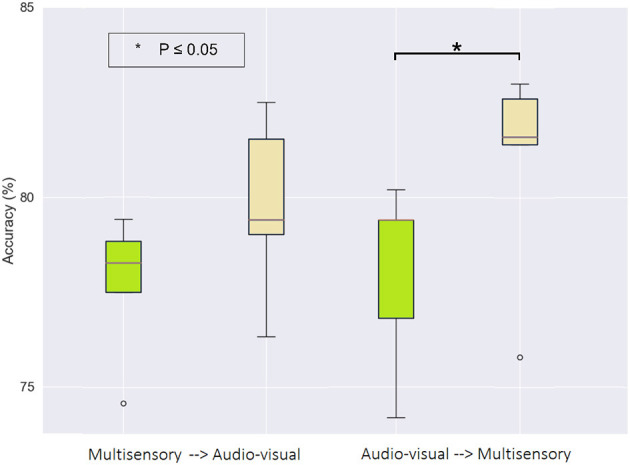
Box-plot of binary classification accuracy for the first (green) and last (beige) motor imagery sessions grouped by motor priming order. The ^*^ symbol indicates a statistically significant difference across experimental conditions using a paired sample t-test.

More importantly, we were interested in investigating which priming condition performed directly before the motor imagery task resulted in the greatest improvement. To this end, Figure 7 illustrates the accuracy achieved in the first (green) and last (beige) MI task, but now separated based on the subjects that performed the multisensory MP task first, followed by audio-visual (left graph), versus those that performed the audio-visual MP first and the multisensory last (right graph). The S-W test only revealed non-normal distribution for values of the first and last MI sessions in the group that received multisensory MP as their second condition. Using a Wilcoxon signed-rank test, a statistically significant difference was observed between the first (M = 78.02, SD = 2.48) and the last (M = 80.89, SD = 2.91) MI session (Z = 2.023, *p* = 0.043) in this group. This finding highlights the potential of multisensory MP directly preceding motor imagery as an interesting candidate to enhance MI-BCI accuracy. This short-term effect of priming on MI accuracy was also reported by Sun et al. (2022).

**Figure 8:**
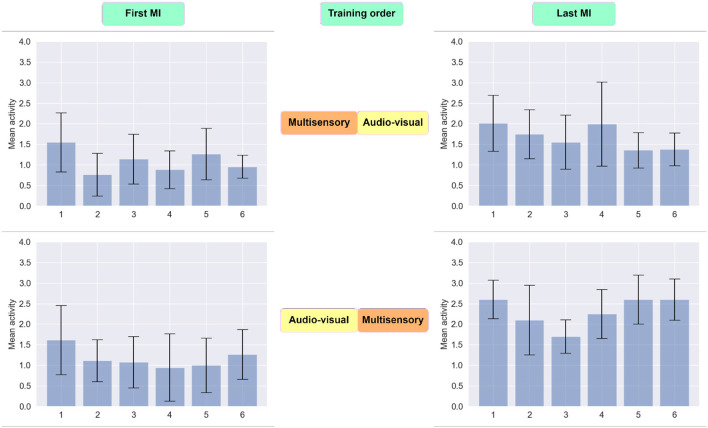
Distribution of extracted data using six CSP filters. The first row reflects the average activity of newly spatially filtered data during the first and last MI session for participants who received multisensory MP prior to audio-visual experience. In the second row, the average activity of newly spatially filtered data are reflected for subjects who exposed to audio-visual MP as their first priming condition.

To gain further insights on the impact that priming has on motor imagery detection accuracy, we explored the impact of priming on the magnitude of the six CSP filters, where greater activity could be indicative of higher discriminability between the two classes. As shown in Figure 8, in both cases the CSP activity was higher in the last MI session relative to the first. This is expected given the effect of training and experience on BCI efficiency (Jochumsen et al., 2018). Notwithstanding, when multisensory MP was performed last, the CSP activity seen during the subsequent MI task was higher than when the audio-visual priming was performed last. This higher CSP activity corroborated the higher overall MI detection accuracy reported previously.

### 3.3. Effect of window size and time-from-cue on accuracy

The results reported above relied on CSP features computed over the entire 10-s imagery window and these features were used for offline BCI analysis. For real-time applications, however, it may be interesting to rely on lower window durations. To this end, we explored the effect that this window size has on overall accuracy. Lower window sizes would mean CSP filters would be computed faster and decisions could be made more quickly. Window size duration corresponds to the amount of time from the task cue.

**Figure 9:**
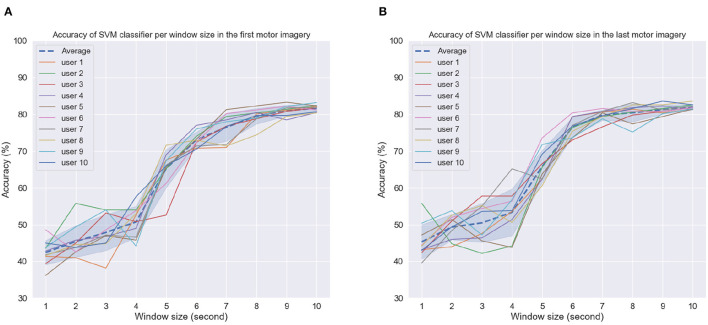
Accuracy as a function of window size per subject in the **(A)** first motor imagery session and **(B)** last motor imagery session. Dashed line indicates average accuracy across users per window length while shaded interval shows the standard deviation.

Figures 9A, B depict the achieved accuracy as a function of window size for the first and last MI tasks, respectively. Accuracy is reported per subject, as well as averaged across subjects (dashed line). As can be seen, for window sizes between 1 and 4 s, chance or below-chance levels were achieved on the unseen test dataset, suggesting that the imagery task should be performed for at least 5 s. However, this could be related to the time users needed to perform the MI task proposed here, i.e., reaching, grabbing, and squeezing the orange. In the first MI task, accuracy levels stabilized for some subjects for window sizes greater than 8 s, whereas in the last MI session, this could be achieved for most subjects around 7 s and for some even at 6 s. These findings suggest that after priming, peak MI-BCI accuracy could be achieved potentially 2 s faster than without priming for some subjects.

**Figure 10:**
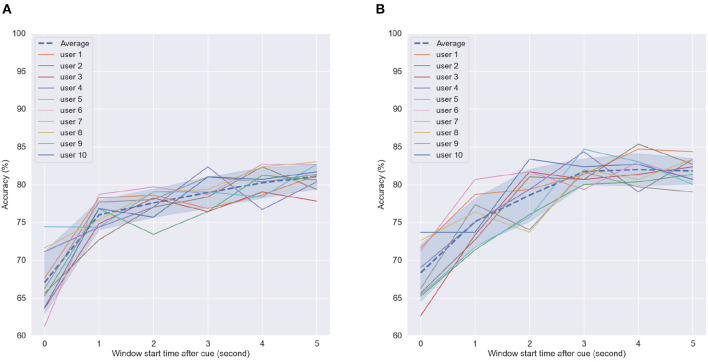
Accuracy of SVM classifier using 5-s fixed-length window size with different start points after cue in **(A)** First motor imagery session, **(B)** Last motor imagery session. Dashed line indicates average accuracy across users per window length while shaded interval show standard deviation.

Next, we examined the effect of time-from-cue on overall accuracy. Time from cue indicates the amount of time waited once the subjects were cued to perform the task until CSP computation was performed. As the motor imagery task was fairly long (reach, grab an orange, move to the middle of the table, and then squeeze), certain parts of the imagery task may elicit stronger sensorimotor cortical activity. A longer time-from-cue duration will likely focus more on the imagery of moving the orange to the middle of the table and squeezing and less about the reaching of the arm to grab the orange. For this analysis, we kept the window size constant and varied the starting point for analysis. A 5-s window length was used based on the previous analysis. Figure 10 shows the achieved accuracy per subject and averaged across all subjects (dashed line) as a function of time-from-cue in seconds.

As can be seen, the greatest gains were seen when 1 s or more were considered post cue presentation for CSP feature computation; such findings corroborate those reported previously in the literature (e.g., Blankertz et al., 2007). For the first MI task, average accuracy continued increasing, whereas in the last MI task, it plateaued after 3 s, where it reached peak values. These findings suggest that the arm reaching part done at the beginning of the imagery task may not generate sufficiently discriminative CSP patterns for the BCI processing pipeline. In fact, this part during priming received no tactile or olfactory stimuli. These only appeared later in the task during the grabbing and squeezing of the oranges. These findings further motivate the use of multisensory MP prior to MI.

### 3.4. Global MI-BCI accuracy

Results reported up to now have been based on classifiers trained and tested on the same subject, thus were fine-tuned on their unique neural patterns. Here, we trained a global classifier where data from all subjects were pooled together. The first row of Table 2 reflects the average accuracy achieved on the test set for the first and last MI sessions. A paired sample t-test suggested a statistically significant difference [*t*(199) = –18.213, *p* ≤ 0.001].

**Table 2:** Global performance accuracy achieved once all left or right oranges were grouped together (binary), vs. when the classification was done per distance level.

**Classification mode**	**First MI accuracy %**	**Last MI accuracy %**
Binary*	79.1 ± 1.33	81.3 ± 1.05
Level 1*	74.3 ± 2.23	73.7 ± 2.38
Level 2*	75.9 ± 2.12	77.1 ± 1.86
Level 3*	73.4 ± 2.05	75.3 ± 1.94

The values reported indicate the average test set accuracy and standard deviation (SD). An asterisk indicates a statistically significant difference across experimental conditions using a paired sample t-test.

Next, we were interested in observing if the distance of the imagined movement had an effect on overall MI-BCI accuracy. In this case, the left versus right classification task was performed three times, once for plates closest to the participant (level 1), at middle distance (level 2), and furthest away (level 3), each comprised of 100 trials. As can be seen, for level 1 classification, the last MI session actually achieved slightly lower accuracy compared to the first MI session. For the other two levels, higher accuracy was achieved during the last MI task. This finding may be explained by the underlying effects of *distance* postulated by Fitt's law in previous studies (e.g., Lorey et al., 2010; Anema and Dijkerman, 2013; Gerig et al., 2018; Errante et al., 2019). For instance, the work by Decety and Jeannerod (1995) showed a linear relationship between time elapsed to imagine a movement with width and distance to the cues. This influence could be a result of spatial presence in virtual environments (Ahn et al., 2019). Therefore, alongside duration and type of stimulus, the distance to the cued object from the participants or the depth of the 3D objects in the virtual environment could potentially play an important role in MI performance. Further studies are needed to validate this hypothesis.

### 3.5. EMG activity

**Figure 11:**
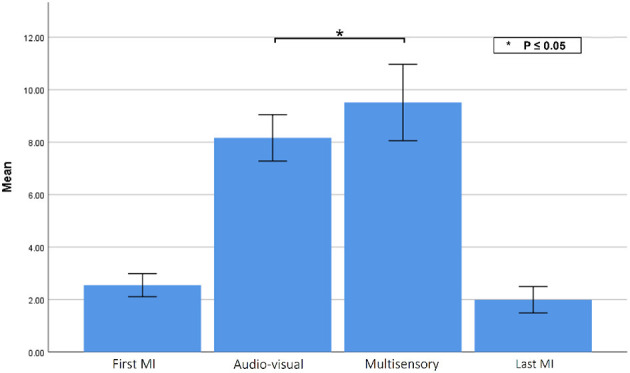
EMG MAV during each experimental condition. Asterisks indicate statistically significant difference.

Lastly, to gauge the changes in EMG activity during the tasks, Figure 11 illustrates the changes in EMG MAV during the two MI tasks, as well as the two priming conditions. Using a paired sample *t*-test, a statistically significant difference was observed [*t*(9) = 2.935, *p* = 0.016] in muscle activity between the audio-visual (M = 8.16, SD = 1.40) and multisensory (M= 9.51, SD= 2.30) priming sessions, as the force feedback gloves were on only in the latter condition. The slight activity observed during the motor imagery tasks are likely indicative of the signal noise floor of the EMG sensors, but could also be an indicator of covert motor function that has been reported during motor imagery (Lebon et al., 2008; Dos Anjos et al., 2022).

### 3.6. Interviews and user feedback

As mentioned previously, at the very end of the experiment an open-ended interview was conducted with each participant. All of the participants reported being able to perceive and recognize the olfactory and tactile stimuli in the multisensory condition. All confirmed they could feel the texture of the objects in the virtual environment and that this helped improve their sense of presence and the realism of the interaction. Regarding the citrus scent, some participants reported it as pleasant, while others suggested it was too intense. Such variability could be associated with age and/or gender related factors, as reported previously by Murray et al. (2013). The majority reported that the olfactory stimulus helped them enhance their sense of immersion, thus corroborating previous studies (Kreimeier et al., 2019; De Jesus et al., 2022).

### 3.7. Limitations and future directions

The presented study was conducted during the sixth wave of the COVID-19 pandemic, therefore, several limitations were applied to ensure participant safety. Consequently, this limited our sample size, as well as the generalizability of the achieved results. As such, future work should aim to increase the participant pool size. Nevertheless, the observed significant changes already corroborate those found in previous studies with higher number of participants, thus the obtained results are promising. Future work should take advantage of multisensory priming directly preceding motor imagery tasks to improve MI-BCI performance, especially for inefficient users. Moreover, analysis of the impact of different window duration during the MI task suggested that for some subjects, decisions could be made up to 2 s faster. These benefits could lead to BCI protocols that are faster and more engaging for participants.

The methods used here relied on conventional feature extraction (i.e., CSPs) and classification (i.e., SVM) pipelines. Future work could explore the use of more recent algorithms, such as deep neural networks. In fact, deep learning approaches have provided opportunities for real-time analysis and interpretation of brain signals (Cho et al., 2021), as well as improved MI accuracy (Zhang et al., 2019), thus could further benefit inefficient users.

The development of the virtual environment for the MP task was driven by the available functionalities of the haptic glove and the available smells within the OVR kit, which was custom-developed for nature scenes. Therefore, future motor priming tasks could benefit from the use of multisensory VR systems that incorporate more realistic scenarios tailored to the preferences and interests of each user. Personalized environments could increase levels of engagement during the MP sessions and potentially further improve MI performance. By providing a more immersive and engaging experience, multisensory VR systems could help users sustain interest in the task at hand. This aspect is particularly important for rehabilitation purposes, where participants have been shown to struggle with motivation and adherence to conventional protocols Oyake et al. (2020). Lastly, the proposed multisensory MP task requires users to have some functional motor control, which may limit its application for individuals with severe motor disabilities, such as those with locked-in syndrome or post-stroke.

## 4. Conclusions

In this paper, we reported the results of a pilot study conducted on 10 participants to evaluate the impact of multisensory motor priming in VR (where olfactory and haptic stimuli were included) to improve motor imagery performance. To this end, a biosensor-instrumented head-mounted display was developed and coupled with an off-the-shelf scent diffuser and haptic glove. Experimental results showed the benefits of a multisensory VR-based priming task relative to a conventional audio-visual VR MP task, with significant improvements achieved in motor imagery detection accuracy. Insights on the impact of window size and time-from-cue duration were also obtained and reported. Overall, these preliminary results provided insights on the advantages of multisensory motor priming on motor imagery performance and offered new perspectives on how to potentially improve MI-BCI performance.

## Data availability statement

The raw data supporting the conclusions of this article will be made available by the authors, without undue reservation.

## Ethics statement

The studies involving human participants were reviewed and approved by Institut National de la Recherche Scientifique (INRS), University of Quebec (number: CER-22-663). The patients/participants provided their written informed consent to participate in this study.

## Author contributions

Both authors listed have made a substantial, direct, and intellectual contribution to the work and approved it for publication.
